# Antidepressant/Anxiolytic and Anti-Nociceptive Effects of Novel 2-Substituted 1,4-Benzodiazepine-2-ones

**DOI:** 10.3797/scipharm.1004-12

**Published:** 2010-05-17

**Authors:** Harjit Singh, Jintana Sattayasai, Pornthip Lattmann, Yodchai Boonprakob, Eric Lattmann

**Affiliations:** 1 School of Life & Health Sciences, Pharmacy, Aston University, Aston Triangle, Birmingham B4 7ET, England; 2 Department of Pharmacology, Faculty of Medicine, Khon Kaen University, 40002 Khon Kaen, Thailand

**Keywords:** Oxazepam, 2-Substituted, 1,4-benzodiazepine, Analgesics, Antidepressants, *In vivo* studies, Dye

## Abstract

Oxazepam (**4a**) has been used as overall starting material in the synthesis of novel 2-substituted 1,4-benzodiazepines.

By reacting Oxazepam **4a** with commercially available hydrazines, hydrazides, semicarbazide, aminoguanidine and *N*,*N*-dimethylamino aniline in ethanol under acetic conditions, a series of diazenyl-1,4-benzodiazepines **5a–5i** and 2-amino-1,4-benzodiazepine **5k** were obtained in good yields.

These novel compounds served as new chemical entities (NCE) for testing in mice. The diazo-benzodiazepine **5d** has shown a promising antidepressant effect in initial experiments *in vivo* at a dose of 5 mg/kg. The highly coloured 2-aminobenzodiazepine derivative **5k** showed over a dose range from 5–50 mg/kg an analgesic effect in mice.

## Introduction

Benzodiazepines are primary agents to treat anxiety and they are the most successful class of chemical compounds worldwide. Around 50 benzodiazepine derivatives have been marketed worldwide. They are all classed as anxiolytics, anticonvulsants, sedatives and muscle relaxants. They mainly act by binding to a specific regulatory site on the GABA_A_ (γ-amino butyric acid) receptor, thus increasing the inhibitory effect of GABA [1].

Since the discovery of benzodiazepines as anxiolytics in the 1960’s, the classical structures of this class of compounds have been widely varied, resulting in benzodiazepine ligands that bind to specific subtypes of the GABA_A_ receptors.

Inspired by the discovery of Asperlicin [2] that contains a tryptophan and benzodiazepine moiety, the 3-amido- and 3-ureido-1,4-benzodiazepin-2-ones have been developed as potent and selective cholecystokinin subtype receptor ligands (CCK-A [3] and CCK-B [4]), respectively.

N_1_ substituted 1,4-benzodiazepines served as lead structure for bradykinin ligands [5], while 4-substituted ureidobenzodiazepines have been modified into neurokinin receptor antagonists [6].

**Fig. 1.**
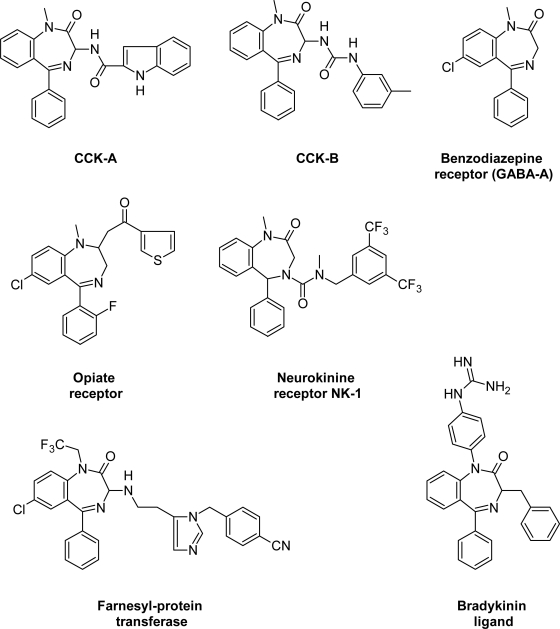
Biologically active benzodiazepines.

Tifluadom [7], is a 2-substituted 1,4-benzodiazepine, binding to the opiate receptor and represents a well known μ receptor antagonist [8]. In a recent patent application, a series of 3-amino-1,4-benzodiazepine were claimed as CCK-antagonists [9]. Further, 1,3-substituted 1,4-benzodiazepine templates were developed into farnesyl-protein transferase inhibitors, which represent potentially useful anticancer agents [10] (Figure 1).

As shown, modified benzodiazepines are uniquely fruitful in the discovery of new lead structures and drugs. Therefore, novel 2-substituted 1,4-benzodiazepines had been synthesised in high yields from 3-hydroxy-1,4-benzodiazepine (oxazepam) and have been subsequently evaluated in standard CNS animal models.

## Results and discussion

### Synthesis

The starting material towards the synthesis of 3-substituted 1,4-benzodiazepines was oxazepam [11], which had been previously prepared by various synthetic methods [12]. Oxazepam has been derived from diazepam [13], by subsequent oxidation of the 3-position [14]. The most common synthesis is the rearrangement of the N-oxide of diazepam via the acetate into oxazepam [15]. We found the most appropriate route towards the synthesis of oxazepam on a multigramme scale based on the oxime **2**, which was converted into the chloride **3** and cyclised in a one pot reaction into the desired oxazepam **4a** or the oxazepam salt **4b** under strictly monitored reaction conditions [16] (Scheme 1).

Mechanistically, the one pot reaction followed the Polonovski Rearrangement [17], in which a benzodiazocrine, an eight membered hetrocyclic ring system, was formed in situ.

A mixture without a major product was formed, when oxazepam was refluxed with amines in ethanol/acetic acid as prescribed by Kulkarmi [18]. 3-Substituted 1,4-benzodiazepines [19] were formed via the 3-chlorinated intermediates, which acted as CCK antagonists and their in vivo evaluation was recently published [20].

The reaction of **4a** under reflux conditions worked well with hydrazines and hydrazides as nucleophiles and furnished the unexpected 2-diazobenzo-diazepines **5a–5g** in good yields, as highly coloured crystalline compounds.

Using semicarbazide or aminoguanidine resulted in the formation of **5h** or **5i** (Table 1).

**Fig. 2.**
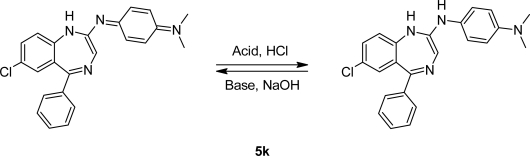
Organic dye

Dimethylaminoaniniline is the only working aniline and formed by this route the 2-amino derivative **5k**. It is violet in alkaline media and turns yellow, after the addition of acid. This novel compound might be useful as an organic dye in fluorescence screening assays for analgesics (Figure 2).

**Fig. 3.**
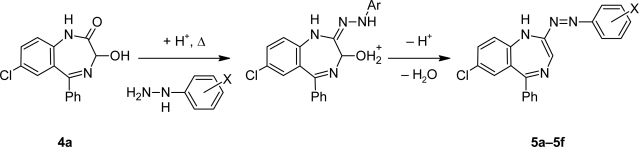
Proposed mechanism for the formation of diazo-benzodiazepines.

A mechanism for the formation of diazenylbenzodiazepines **5a–5i** is proposed. The nucleophilic attack of the substituted phenylhydrazines formed the hydrazones of **4a**, and under acetic conditions the intermediate formed in a condensation reaction the diazo-derivatives **5a–5i** (Figure 3).

### Animal studies

Using standard CNS assays the anxiolytic, antidepressant and analgesic properties were systematically evaluated in mice. The elevated x-maze and the light/dark box were applied to test for anxiolytic activity. Anilino-benzodiazepine **5k** showed a low CCK binding activity and this was not further investigated as CCK antagonists previously developed from the 3-substituted series had a better potency [20]. The tail suspension and the forced swim test served as assays for antidepressant activity. The diazo series **5d–5f** was found different from the control from 5 mg/kg onwards and the dinitro- derivative **5f** displayed unwanted side effects as accessed by the rota rod and the wire mesh grasping test. These two assays were added to the screening to identify mainly unwanted muscle relaxing properties or an impairment of coordination. Considering these properties and the presence of toxicophore, a nitrogroup, the chlorinated diazo- derivative **5d** was forwarded to further evaluation. The thermal tail flick test (tail immersion test) and the hot plate test were applied to investigate the analgesic effects of the 2-substituted benzodiazepines (Table 2).

The 2-aniline derivative **5k** showed promising activity from 5 mg/kg and it was tested further in the tail flick test for a range of doses.

**Fig. 5.**
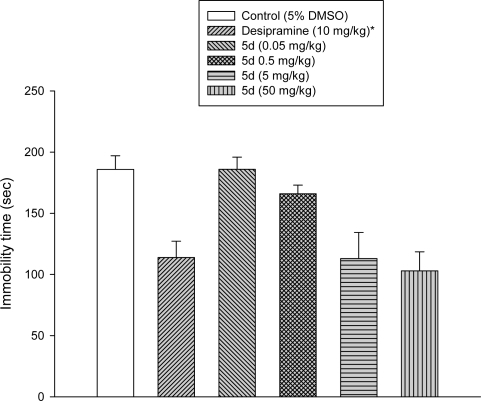
Dose-effect relationship of **5d** on the immobility time for the forced swim test in mice.

The diazo-benzodiazepine **5d** was tested further in the forced swim test for a dose range from 0.05–50 mg/kg. In presence of the standard antidepressant desimpramine (10 mg/kg) the immobility time was reduced from 180s to 120s. Dose dependant a reduction was found for **5d** with a comparable potency and magnitude to desipramine (Figure 5).

**Fig. 6.**
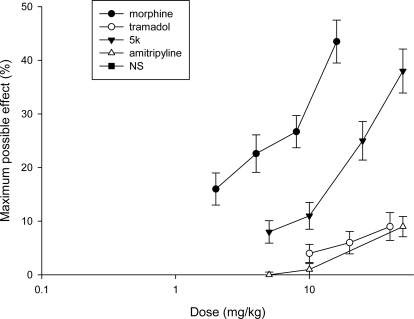
MPE in the tailflick test for compound **5k** compared with morphine, tramadol and amitriptyline.

The analgesic effect of **5k** was tested in the tail flick test from 5–50 mg/kg in comparison with morphine, tramadol and amitriptyline as standards and the maximum possible effects are shown in Figure 6.

It appears that the activity is opiate like and in vitro studies are ongoing. 3-Anilinobenzodiazepine based CCK antagonists [21] such as the *N*-methylanilino-benzo-diazepine potentiated the analgesic effect of morphine, while the 2-substituted benzo-diazepine **5k** showed a dose dependent analgesic effect on its own.

## Conclusions

By using simply refluxing oxazepam with hydrazines and hydrazides in the presence of acid novel 2-diazo-1,4-benzodiazepines were furnished, of which the diazo derivatives and **5k** were highly coloured. These new chemical structures were identified as new lead structures for antidepressents, anxiolytics and analgesics. Further studies to explore the underlying biological mechanism are currently being evaluated and the colour/fluorescence of the agents may be additionally useful in pharmacology to locate the distribution of receptors.

## Experimental

### Chemistry

The chemicals were purchased from Aldrich, UK, and Lancaster Synthesis, UK. Mass-Spectras were obtained by Atmospheric Pressure Chemical Ionisation (APCI), using a Hewlett-Packard 5989b quadrupole instrument connected to an electrospray 59987A unit, with automatic injection (Hewlett-Packard 1100 series autosampler). Samples were dissolved in HPLC grade methanol or acetonitrile. Both proton and carbon NMR spectra were obtained on a Brucker AC 250 instrument, calibrated with the solvent reference peak. Infra-red spectra were plotted from KBr discs on a Mattson 300 FTIR Spectrophotometer. Melting points were recorded from a Stuart Scientific Melting Point (SMP1). Analytical Thin Layer Chromatography was obtained using aluminium sheets, silica gel_60_ F254, 250 nm and were visualized using ultraviolet light.

### (2-Amino-5-chlorophenyl)(phenyl)methanone oxime (2)

2-Amino-5-chlorobenzophenone (160 mmol, 37.08 g) and hydroxylamine HCl (320 mmole, 11.12 g) were placed in a dry round bottom flask. Ethanol (230 ml) and pyridine (50 ml) were added and the mixture was refluxed for 72 hrs. The solution was allowed to cool and one third of the solvent was removed.

(A): The remaining residue was partitioned first with ether then water. The organic phase was washed with water and dried over magnesium sulphate and then concentrated to dryness. The yellow solid was heated with a minimum amount of toluene and then cooled to room temperature and allowed to crystallise.

(B): The remaining residue was precipitated with water. The solid was filtered, washed with water and then heated with a minimum amount of toluene and then cooled to room temperature and allowed to crystallise. 89% (including second crop); IR (KBr) cm^−1^: 3375, 3020, 3400, 2950, 1600, 825; MF C_13_H_11_N_2_OCl; MW 246.7; MS (APCI (+)): 247, 249 (M+1), 229, 231 (−H_2_O) m/z; ^1^H NMR (DMSO-d_6_) 300K : 11.57 (s, OH), 7.37 (m, Phenyl-H), 6.80 (s, C3-H), 7.13 (d, C_5_-H, J=6.3 Hz) 6.77 (d, C_6_-H, J=5.3 Hz) 4.80 (s, NH) ppm; ^13^C NMR (DMSO-d_6_) 300K, 156.6 (C=N), 149.2 (C-NH_2_), 116.7, 132.7, 130.9, 130.0, 128.6, 128.0, 127.8, 126.9, 121.1, 127.1, (Ar-C) ppm.

### 2-Chloro-N-{4-chloro-2-[(hydroxyimino)(phenyl)methyl]phenyl} acetamide (3)

A solution of 2-amino-5-chlorophenyl)(phenyl)methanone oxime (**2**, 142 mmol, 35.18 g) in ether (1000 ml) and water (300 ml) was stirred in an ice bath at 0–5°C. Chloroacetyl chloride (160 mmol, 12.8 ml) was added dropwise, over 30 mins, whilst maintaining a slightly basic solution with the addition of 15% aqueous sodium hydroxide. After the addition of chloroacetyl chloride the reaction was stirred for an additional 3 hrs at room temperature. The organic phase was washed with water, dried over magnesium sulphate and concentrated to dryness to yield a white powder. 94%; IR (KBr) cm^−1^: 3390, 3300, 3015, 2900, 1660, 820; MF C_15_H_12_N_2_O_2_Cl_2_; MW 323.2; MS (APCI(+)): 323, 325 (M+1), 305, 307 (−H_2_0) m/z; ^1^H NMR (DMSO-d_6_) 300K : 11.92 (s, N- OH), 9.25 (s, NH) 7.39 (s, Phenyl-H), 7.20 (s, C3-H), 7.53 (d, C6-H, J=8.7 Hz) 7.83 (d, C5-H, J=8.8 Hz) 4.06 (s, -CH_2_-) ppm; ^13^C NMR (DMSO-d_6_) 300K, 157.2 (C=N-OH), 137.1 (C-NH-),43.1 (-CH_2_-), 136.9, 136.0, 133.9, 133.1,132.8, 132.2, 130.8, 130.1,127.9, 125.8, 124.9 (Ar-C) ppm.

### 7-Chloro-3-hydroxy-5-phenyl-1,3-dihydro-2H-1,4-benzodiazepin-2-one (Oxazepam, 4a)

A solution of 2-chloro-*N*-{4-chloro-2-[(hydroxyimino)(phenyl)methyl]phenyl}acetamide (**3**, 134 mmol, 43.28g) in ethanol (900 ml) and sodium hydroxide (2M, 280 ml) were stirred at room temperature over night. The precipitate that formed was separated by filtration and dissolved in a minium amount of ethanol, water 60:40 mix, (undissolved oxazepam salt was collected and dried). The mixture was acidified to pH 1.0–2.0 by the addition of concentrated hydrochloric acid. The filtrate was cooled in a ice bath to 0°C–10°C over night. The precipitate was filtered and dried to give the crude product (light brown solid). 59.8% (crystallisation with ethanol or 1,4 dioxane); IR (KBr) cm^−1^: 3385, 3025, 3320, 3010, 1705, 1590, 730; MF C_15_H_11_N_2_O_2_Cl; MW 286.7; MS (APCI(+)): 287, 289 (M+1), 269, 271 (−H_2_O) m/z; ^1^H NMR (DMSO-d_6_) 300K : 10.81 (s, N-H), 7.48 (s, Phenyl-H), 7.23 (s), 7.25 (d, Ar-H, J= 8.8 Hz), 7.64 (d, Ar-H, J= 8.7 Hz), 6.33 (d, OH, J= 8.7 Hz), 4.78 (d, C3-H, J= 8.7 Hz) ppm; ^13^C NMR (DMSO-d_6_) 300K : 170.3 (C=N), 165.9 (C=O), 123.7, 127.1, 128.3, 128.5 (2xC), 128.9, 129.7, 129.8 (2xC), 131.0, 132.3, 138.5 (Ar-C), 83.3 (CH-OH) ppm.

### Sodium 7-chloro-3-hydroxy-5-phenyl-3H-1,4-benzodiazepin-2-olate (oxazepam salt, 4b)

22.3%; IR (KBr) cm^−1^: 3350, 3030, 2950, 1690, 1590, 1205, 760; MF C_15_H_11_N_2_O_2_Cl; MW 286.7; MS (APCI(+)): 287, 289 (M+1), 269, 271 (−H_2_O) m/z; ^1^H NMR (DMSO-d_6_) 300K : 7.87 (m, Phenyl–H), 7.30 (d, Ar-H, J= 8.8 Hz), 6.97 (s, Ar-H), 6.94 (d, Ar-H, J= 8.6 Hz), 5.90 (s, C-OH), 4.25 (s, C3-H) ppm; ^13^C NMR (DMSO-d_6_) 300K : 194.0 (C=N), 168.7 (C=O), 122.7, 126.5, 127.2, 128.7 (2xC), 129.2, 129.6, 129.9 (2xC), 131.3, 132.8, 139.5 (Ar-C), 83.5 (CH-OH) ppm.

### General synthesis of 7-chloro-5-phenyl-1, 4-benzodiazepines 5a–5i, 5k

A solution of oxazepam (**4**, 0.1 g, 3.5x10^−4^ mol) in ethanol (10 ml) and 3 drops of glacial acetic acid were refluxed for 20–25 hours with the appropriate amine (3.56x10^−4^ mol). The progress of the reaction was monitored by TLC with diethyl ether. After complete reaction the precipitate was filtered, washed with ethanol (twice) and dried.

#### 7-Chloro-5-phenyl-2-[(*E*)-phenyldiazenyl]-1*H*-1,4-benzodiazepine (**5a**)

Yield: 65.0%, mp: 178°C. Mol. Weight: 393.3 Mol. Formula: C_21_H_14_Cl_2_N_4_, MS (APCI(+)): 393, 395 (M+1) m/z. IR (KBr-disc) max: 3436, 2929, 1611, 1490, 1390, 1280, 1143, 1085, 823, 703 cm^−1. 1^H NMR (DMSO-d_6_) 300K : 11.30 (s, NH), 8.11 (s, C3-H), 8.01–8.10 (d, Ar-H, J= 8.8 Hz), 7.99–8.04 (dd, Ar-H, J= 9.0 Hz), 7.90 (s, Ar-H), 7.77–7.85 (m, phenyl-2H), 7.65–7.69 (m, phenyl-3H), 7.30-7.35 (d, Ar-2H, J=8.9 Hz), 7.15–7.18 (d, Ar-2H, J=8.9 Hz) p.p.m. ^13^C NMR (DMSO-d_6_) 300K : 124.1, 126.0, 126.9 (2xC), 128.3, 128.5, 129.3 (2xC), 130.4, 130.6 (2xC), 132.1 (2xC), 132.2, 133.5, 133.8, 143.7, 150.2 (Ar-C), 152.8 (C3), 152.9 (C-N=N), 169.2 (C=N) p.p.m.

#### 7-Chloro-2-[(*E*)-(4-methylphenyl)diazenyl]-5-phenyl-1*H*-1,4-benzodiazepine (**5b**)

66.0%; mp: 182°C; IR (KBr) cm^−1^: 3428, 3060, 2923, 2364, 1560, 1519, 1390, 1253, 811, 699; MS (APCI(+)): 373, 375 (M+1) m/z; MF C_22_H_17_ClN_4_; MW 372.8; ^1^H NMR (DMSO-d_6_) 300K : 13.86 (s, NH), 8.38–8.41 (dd, Ar-H, J=9.0 Hz), 8.08–8.14 (dd, Ar-H, J=9.0 Hz), 7.96 (s, Ar-H), 7.78–7.85 (m, phenyl-2H), 7.62–7.69 (m, phenyl-3H), 7.42 (s, C3-H), 7.26–7.30 (d, Ar-2H, J=8.4 Hz), 7.13–7.17 (d, Ar-2H, J=8.2 Hz), 2.23 (s, CH_3_) ppm; ^13^C NMR (DMSO-d_6_) 300K: 19.5 (CH_3_), 126.2, 126.4 (2xC), 128.1 (2xC), 129.2, 129.4, 131.3 (2xC), 130.6, 131.2 (2xC), 132.8, 132.9, 133.3, 133.6, 149.0, 149.7 (Ar-C), 149.9 (C3), 158.8 (C-N=N), 167.2 (C=N) ppm.

#### 7-Chloro-2-[(*E*)-(3,4-dimethylphenyl)diazenyl]-5-phenyl-1*H*-1,4-benzodiazepine (**5b**)

61.0%; mp: 198°C; IR (KBr) cm^−1^: 3443, 2921, 2856, 2364, 1569, 1517, 1382, 1261, 832, 699 ; MS (APCI(+)): 387, 389 (M+1) m/z; MF C_23_H_19_ClN_4_; MW 386.9; ^1^H NMR (DMSO-d_6_) 300K : 13.85 (s, NH), 8.37–8.45 (dd, Ar-H, J=8.9 Hz), 8.05–8.14 (dd, Ar-H, J=9.0 Hz) 8.01–8.08 (d, Ar-H, J=8.9 Hz), 7.97 (s, C3-H), 7.79–7.91 (m, phenyl-2H), 7.62–7.74 (m, phenyl-3H & overlapping Ar-H), 7.41 (s, Ar-H), 7.20 (s, Ar-H), 2.25 (s, CH_3_), 2.18 (s, CH_3_) ppm; ^13^C NMR (DMSO-d_6_) 300K: 19.3 (CH_3_), 20.2 (CH_3_), 121.4, 122.0, 125.9, 127.5, 128.2, 129.2 (2xC), 129.3, 129.9 (2xC), 130.3, 130.3, 130.7, 132.9, 133.0, 134.6, 142.7, 148.7 (Ar-C), 150.2 (C3), 159.3 (C-N=N), 167.8 (C=N) ppm.

#### 7-Chloro-2-[(*E*)-(4-chlorophenyl)diazenyl]-5-phenyl-1*H*-1,4-benzodiazepine (**5d**)

65%; mp: 182°C; IR (KBr) cm^−1^: 3436, 2929, 1611, 1490, 1390, 1280, 1143, 1085, 823, 703; MS (APCI(+)): 393, 394, 395 (M+1) m/z; MF C_21_H_14_Cl_2_N_4_; MW 393.3; ^1^H NMR (DMSO-d_6_) 300K : 11.30 (s, NH), 8.11 (s, C3-H), 8.01–8.10 (d, Ar-H, J= 8.8 Hz), 7.99–8.04 (dd, Ar-H, J= 9.0 Hz), 7.90 (s, Ar-H), 7.77–7.85 (m, phenyl-2H), 7.65–7.69 (m, phenyl-3H), 7.30–7.35 (d, Ar-2H, J=8.9 Hz), 7.15–7.18 (d, Ar-2H, J=8.9 Hz) ppm; ^13^C NMR (DMSO-d_6_) 300K : 124.1, 126.0, 126.9 (2xC), 128.3, 128.5, 129.3 (2xC), 130.4, 130.6 (2xC), 132.1 (2xC), 132.2, 133.5, 133.8, 143.7, 150.2 (Ar-C), 152.9 (C3), 152.9 (C-N=N), 169.2 (C=N) ppm.

#### 7-Chloro-2-[(*E*)-(4-nitrophenyl)diazenyl]-5-phenyl-1*H*-1,4-benzodiazepine (**5e**)

67.0 %; mp: 198°C; IR (KBr) cm^−1^: 3440, 3290, 2915, 2360, 1620, 1505, 1330, 1320, 1135, 1085, 830; APCI(+): 449, 451 (M+1) m/z; C_21_H_13_ClN_6_0_4_; MW 448.8; ^1^H NMR (DMSO-d_6_) 300K : 11.96 (s, NH), 8.82 (d, Ar-H, J= 2.7 Hz), 8.43 (dd, Ar-H, J= 9.5 Hz), 8.14 (d, Ar-H, J= 9.1 Hz), 8.07 (d, Ar-H, J= 7.7 Hz), 8.05 (d, Ar-H (overlapping), J= 8.9 Hz), 7.93 (d, Ar-H, J= 2.1 Hz), 7.83–7.87 (m, phenyl-2H), 7.66–7.70 (m, phenyl-3H) ppm. ^13^C NMR (DMSO-d_6_) 300K : 120.9, 121.8, 122.5, 124.7, 125.1 (2xC), 127.9, 128.5, 130.3, 131.2, 131.7 (2xC), 132.6, 133.8, 134.9, 136.4, 140.2 (Ar-C), 144.6 (C3), 153.5, 154.3 (C-N=), 168.0 (C=N) ppm.

#### 7-Chloro-2-[(*E*)-(2,4-dinitrophenyl)diazenyl]-5-phenyl-1*H*-1,4-benzodiazepine (**5f**)

70.0 %; mp: 217°C; IR (KBr) cm^−1^: 3445, 3195, 3035, 1595, 1580, 1560, 1530, 1500, 1340, 1280, 1265, 1150, 1110; MS (APCI(+)): 404, 406 (M+1) m/z; MF C_21_H_14_ClN_5_0_2_; MW 403.8; ^1^H NMR (DMSO-d_6_) 300K : 11.86 (s, NH), 8.24 (s, Ar-H), 8.19 (d, Ar-2H, J= 9.3 Hz), 8.12 (s, C3-H), 8.04 (dd, Ar-H, J= 9.0 Hz), 7.92 (d, Ar-H, J= 2.3 Hz), 7.81–7.85 (m, phenyl-2H), 7.67–7.69 (m, phenyl-3H), 7.26 (d, Ar-H, J= 9.2 Hz) ppm; ^13^C NMR (DMSO-d_6_) 300K : 112.7 (2xC), 122.3, 126.0, 126.5 (2xC), 129.3 (2xC), 130.3 (2xC), 130.8, 131.2, 132.7, 135.4, 136.7, 140.0, 140.5, 149.9 (Ar-C), 150.3 (C3), 158.1(C-N=), 168.2 (C=N) ppm.

#### 7-Chloro-5-phenyl-2-[(*E*)-(phenylcarbonyl)diazenyl]-1*H*-1,4-benzodiazepine (**5g**)

41.0%; mp: 204°C; IR (KBr) cm^−1^: 3395, 3060, 2358, 1661, 1560, 1531, 1474, 1386, 1264, 1131, 1074, 835, 701; MS (APCI(+)): 387, 388, 389 (M+1) m/z; MF C_22_H_15_ClN_4_O; MW 386.8; ^1^H NMR (DMSO-d_6_) 300K : 12.28 (s, NH), 8.69 (s, C3-H), 8.17–8.21 (d, Ar-H, J= 8.9 Hz), 8.07–8.11 (dd, Ar-H, J= 9.0, 8.9 Hz), 7.95–7.99 (m, phenyl-2H), 7.81–7.85 (m, phenyl-3H), 7.53–7.69 (m, Ar-H & s, Ar-H overlapping) ppm; ^13^C NMR (DMSO-d_6_) 300K : 122.7, 126.1 (2xC), 128.4, 129.1, 129.3 (2xC), 130.4 (2xC), 130.5, 130.9, 131.5, 131.6, 133.4 (2xC), 133.6, 135.7, 136.5 (Ar-C), 150.0 (C3), 158.0 (C-N=N) 168.3 (C=N), 182.8 (C=O) ppm.

#### (*E*)-2-(7-Chloro-5-phenyl-1*H*-1,4-benzodiazepin-2-yl)diazenecarboxamide (**5h**)

50.0 %; mp: 272°C; IR (KBr) cm^−1^: 3430, 2975, 2925, 2360, 1720, 1680, 1585, 1535, 1410, 1390, 1345, 1180, 1100, 835; APCI(+): 326, 328 (M+1), 309, 311 (−NH_2_), 283+ m/z; C_16_H_12_ClN_5_0; MW 325.8; ^1^H NMR (DMSO-d_6_) 300K : 10.89 (s, NH), 8.13 (d, Ar-2H, J= 9.0 Hz), 8.04 (d, Ar-H, J= 2.3 Hz), 7.95 (d, Ar-H, J= 2.1 Hz), 7.80–7.85 (m, phenyl-2H), 7.63–7.67 (m, phenyl-3H), 6.52 (s, NH_2_) ppm; ^13^C NMR (DMSO-d_6_) 300K : 125.3 (2xC), 126.7, 127.1, 130.5, 130.7, 131.0, 131.8 (2xC), 132.3, 136.2, 140.9 (Ar-C), 146.4 (C3), 164.8 (C-N=), 166.1 (C=N), 167.2 (C=O) ppm.

#### (*E*)-2-(7-Chloro-5-phenyl-1*H*-1,4-benzodiazepin-2-yl)diazenecarboximidamide (**5i**)

53.0 %; mp: 243°C; IR (KBr) cm^−1^: 3423, 2923, 2360, 1627, 1519, 1388, 1121, 620; APCI(+): 325, 327 (M+1), 308, 310 (−NH_2_) m/z; C_16_H_13_ClN_6_; MW 324.8; ^1^H NMR (DMSO-d_6_) 300K : 11.04 (s, NH), 8.33 (s, C3-H), 8.08–8.12 (d, Ar-H, J= 9.0 Hz), 8.01–8.06 (dd, Ar-H, J=8.9,9.0 Hz), 7.90 (s, Ar-H), 7.79–7.83 (m, phenyl-2H), 7.64–7.69 (m, phenyl-3H), 7.20 (s, NH_2_) ppm.

#### 7-Chloro-*N*-[4-(dimethylamino)cyclohexa-2,5-dien-1-ylidene]-5-phenyl-1*H*-1,4-benzodiazepin-2-amine (**5k**)

62.0 %; mp: 182–186°C; IR (KBr) cm^−1^: 3430, 2890, 2800, 1625, 1575, 1520, 1475, 1440, 1500, 1350, 1280, 1160, 825; MS (APCI(+)): 387, 389 (M+1) m/z; MF C_23_H_20_ClN_4_; MW 387.9; ^1^H NMR (CDCl_3_) 300K : 8.95 (s, NH), 8.24 (d, Ar-H, J= 9.0 Hz), 8.09 (d, Ar-H, J= 2.3 Hz), 7.86 (dd, Ar-H, J= 9.1 Hz), 7.81–7.84 (m, phenyl-2H), 7.61–7.63 (m, phenyl-3H), 7.55 (d, Ar-H, J= 6.9 Hz), 7.27 (s, C3-H), 6.76 (d, Ar-H, J= 7.0 Hz), 3.04 (s, 2x CH_3_) ppm; ^13^C NMR (CDCl_3_) 300K : 40.3 (2x CH_3_), 112.1 (2xC), 122.8, 123.8 (2xC), 125.9, 128.8 (2xC), 129.9 (2xC), 130.3, 131.3, 133.9, 134.9, 136.5, 138.9, 150.2, 150.5, 153.3 (Ar-C), 158.7 (C-N=), 168.3 (C=N) ppm.

### Animal studies

Experiments were conducted in male IRC mice obtained from the Animal House, Faculty of Medicine, Khon Kaen University. Each experimental group consisted of 6–8 animals and the treatment procedures were approved by the ethical committee, Faculty of Medicine, Khon Kaen University (HO 2434-76) accord with current UK legislation.

Mice were intraperitoneal injected with either test compound dissolved in 5% DMSO at the volume not more than 0.2 ml/animal. At 30 min after treatment, animals were tested as described in the following sections.

### Anxiolytic activity tests

The light/dark box: Mice were placed in the light part of the light/dark box. The box was a plexiglass cage, 25x50x20 cm, having one-third as a dark and two-third as a light compartment. A 40-W light bulb was used and positioned 10 cm above the centre of the light component. The animals could walk freely between dark and light parts through the opening. The time animals spent in light part during the 5 min interval was recorded. The mouse was considered to be in the light part when its 4 legs were in the light part.

The elevated plus-maze: The wooden elevated plus-maze consisted of two open arms (30x10 cm) without any walls, two enclosed arms of the same size with 5-cm high side walls and end wall, and the central arena (10x10 cm) interconnecting all the arms. The maze was elevated approximately 30 cm height from the floor. At the beginning of the experiment the mouse was placed in the central arena facing one of the enclosed arms. During a 5 min interval, the time animals spent in the open arms of plus-maze was recorded. The mouse was considered to be in the open part, when it had clearly crossed the line between the central arena and the open arm with its 4 legs.

### Nociception tests

#### The thermal tail-flick test:

The thermal response latency was measured by the tail flick test. The animals were placed into individual restraining cages leaving the tail hanging freely. The tail was immersed into water preset at 50°C and the response time, at which the animal reacted by withdrawing its tail from water, was recorded.

The thermal response latency was measured by the tail flick test. The base line withdrawal thresholds (BT) were recorded prior to the first injection. Test thresholds (TT) were measured 60 min after the second injection. The cut off time was set to 45s. This was to avoid any tissue damage to the paw during the course of analgesia testing. The test thresholds were expressed as a percentage of Maximal Possible Effect (% MPE) using the equation:

$$\%\text{MPE}=\frac{\text{TT}-\text{BT}}{45-\text{BT}}\cdot 100$$

#### The hot plate test:

Mice were placed on a hot plate that was thermostatically maintained at 50°C. A plexiglass box was used to confine the animal to the hot plate. The reaction time of each animal (either paw licking or jumping) was considered a pain response and the latency to react was recorded.

### Antidepression tests

#### 

##### The tail suspension test:

Mice were hung by their tail on the tail hanger using sticky tape for tail fixation, at approximately 1 cm from the end. The hanger was fixed in the black plastic box (20x20x45 cm) with the opening at the top front. The distance between the hanger to floor was approximately 40 cm. The mouse was suspended in the air by its tail and the immobile time was recorded during the period of 5 min. The duration of immobility was defined as the absence of all movement except for those required for respiration.

##### The forced swim test:

The forced swim test was carried out in a glass cylinder (20 cm diameter, 30 cm height) filled with water to the height of 20 cm. The water temperature was approximately 25–28°C. Mice were gently placed into the water and the immobility time was recorded by an observer during the period of 5 min. Immobility was defined as absence of all movement and remained floating passively in the water with its head just above the water surface.

### Motor activity tests

#### 

##### The rota-rod test:

Mouse was placed on the rotating drum with the acceleration speed (Acceler. Rota-rod, Jones & Roberts, for mice 7650, Ugo Basile, Italy). The time animal spent on the rod is recorded.

##### The wire mesh grasping test:

Mouse was placed on a wire mesh (20x30 cm). After a few seconds, the mesh was turned 180° and the time animal hold on the mesh was recorded.

### Statistical methods

The data were expressed as mean ± SD and one-way analysis of variance (ANOVA) and supplementary Tukey test for pair wise comparison were tested to determine for any significant difference at p< 0.05.

## Figures and Tables

**Sch. 1.**
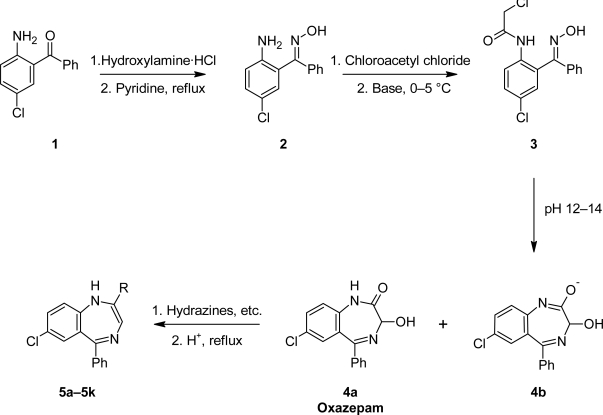
Synthesis of 2-substituted 1,4-benzodiazepines.

**Tab. 1.** Overview of synthesised 2-substituted 1,4-benzodiazepines 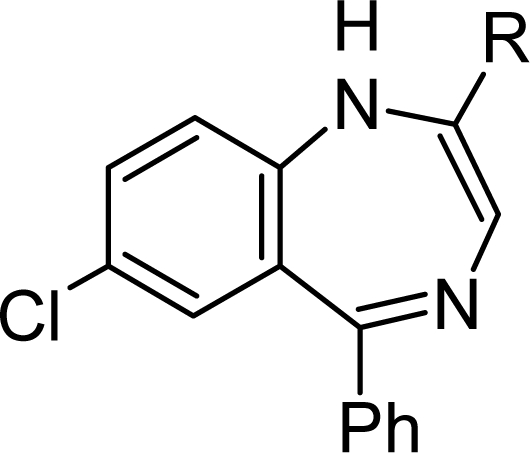

**Entry**	**R**	**Yield %**	**Entry**	**R**	**Yield %**
**5a**	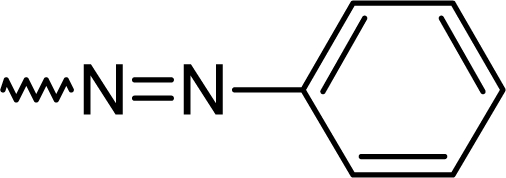	47	**5f**	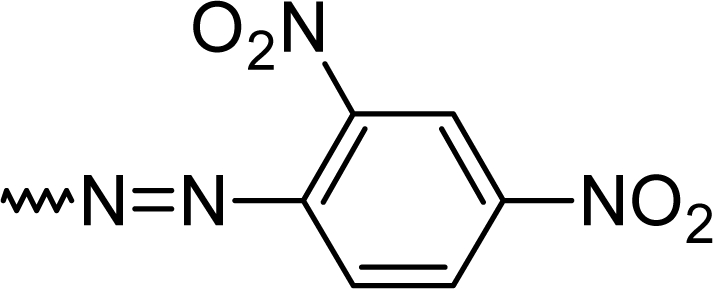	67
**5b**	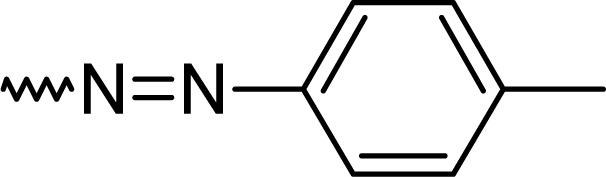	66	**5g**	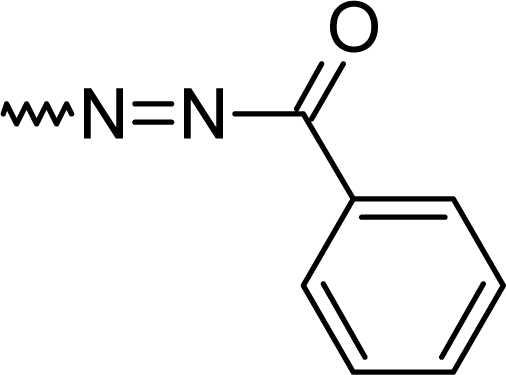	41
**5c**	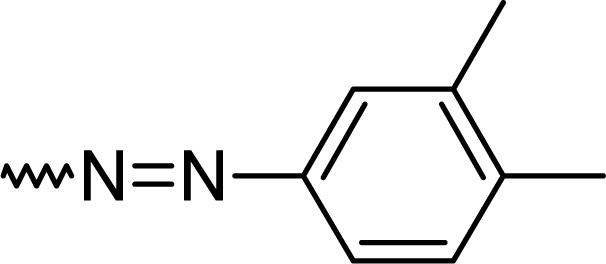	61	**5h**	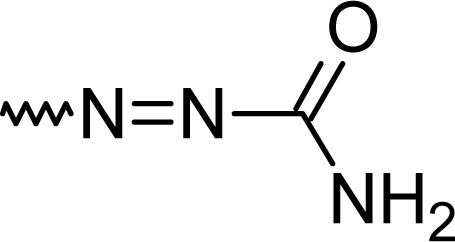	50
**5d**	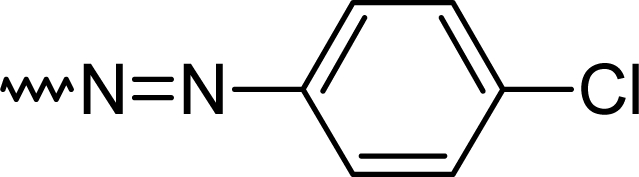	65	**5i**	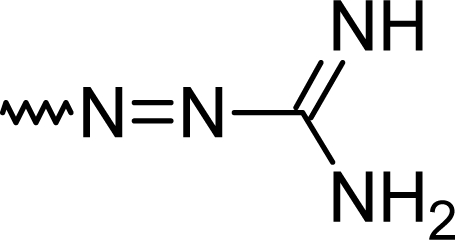	53
**5e**	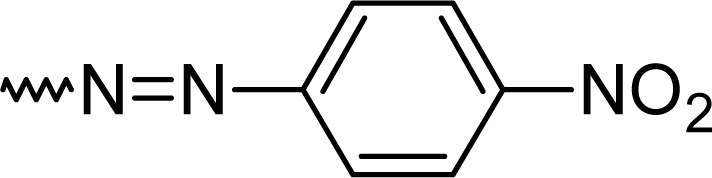	70	**5k**	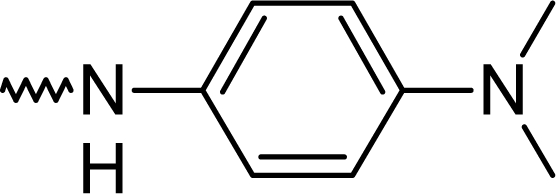	62

**Tab. 2.** *In vivo* evaluation of selected 1,4-benzodiazepines

**Cpd**	**Elevated plus-maze**	**Light/dark box**	**Tail suspendsion test**	**Forced swim test**	**Thermal tail flick test**	**Hot plate test**	**Rota-rod test**	**Wire mesh grasping test**
**5a**	–	–	–	–	–	–	10	10
**5d**	–	–	5	5	10	10	–	–
**5e**	–	–	5	5	10	10	–	–
**5f**	–	–	5	5	10	10	10	10
**5k**	10	10	–	–	5	5	–	–

– … no significance could be observed at 0.1, 0.5, 1.0, 2.5, 5.0 and 10 mg/kg compared to the control;

MED: minimum effective dose [mg/kg] given in above table
